# Environmental Effects of the Livestock Industry: The Relationship between Knowledge, Attitudes, and Behavior among Students in Israel

**DOI:** 10.3390/ijerph16081359

**Published:** 2019-04-16

**Authors:** Keren Dopelt, Pnina Radon, Nadav Davidovitch

**Affiliations:** 1 Department of Public Health, School of Health Sciences, Ashkelon Academic College, Ashkelon 78211, Israel; pnina.gas86@gmail.com; 2 School of Public Health, Faculty of Health Sciences, Ben Gurion University of the Negev, Beersheba 8410501, Israel; nadavd@bgu.ac.il

**Keywords:** environmental pollution, sustainability, livestock industry, pro-environmental behavior, knowledge and attitudes

## Abstract

The livestock industry has numerous and diverse impacts on the environment. In a cross-sectional study using an online questionnaire, 361 students were asked about their knowledge, attitudes, and behavior related to the environmental impact caused by livestock industry. The data were analyzed using correlations, *t*-tests for independent samples, and linear regression models. We found that students have almost no knowledge about the environmental impact of the food they consume, their attitudes are moderately pro-environmental, yet they are not strict about pro-environmental behavior. Students with higher levels of environmental knowledge demonstrated more pro-environmental attitudes and behavior; attitudes mediate the relationship between level of knowledge and behavior with respect to environmental pollution caused by the livestock industry. In addition, participants that rear/reared animals demonstrated more knowledge and pro-environmental attitudes and behavior, and women demonstrated more pro-environmental attitudes and behavior than men. There is a need to raise awareness of the environmental and health impacts caused by livestock industry. An introductory course on environmental science should be integrated into different academic study programs. Further research should be conducted among additional population sectors.

## 1. Introduction

### 1.1. Literature Review

Production of food from animals has accelerated during the last 100 years, in response to growing demand [1]. Throughout the world approximately 70 billion animals are reared as domestic animals annually, with more than 6 million animals killed for food each year [2], and approximately 56 billion mammals and birds slaughtered each year [3]. According to a report by the Food and Agriculture Organization (FAO) of the United Nations, titled, “The Long Shadow of the Animal Industry” [1], global meat consumption doubled during the period 1980–2002. According to future predictions, global meat production is expected to double from 229 million tons in 1999 to 465 million tons by 2050. Milk production is expected to increase from 580 to 1043 million tons [1]. Besides the humane aspects associated with the rearing and slaughtering conditions of animals in the food industry, the great increase in the consumption of animal products has a most severe impact on the environment. The FAO report states that “The meat industry has a marked impact on a general global scale on water, soils, extinction of plants and animals, and consumption of natural resources, and it has a strong impact on global warming” [1].

### 1.2. The Impact of Animal Product Consumption on the Environment

The livestock industry is the source of a broad spectrum of environmental impacts [3]. The first and most important is climate change [4]. In the third chapter of the FAO report [1] it is estimated that 18% of global greenhouse gas emissions are caused by the livestock industry. The amount of carbon dioxide (CO_2_) released to the atmosphere is estimated at approximately 7516 million tons per year [1,3]. According to Goodland and Anhang [5] this estimate is too low. According to their calculations the global livestock industry is responsible for at least 51% of the greenhouse gases emitted to the atmosphere and the amount of carbon dioxide is estimated at 32,564 million tons. This large difference stems partly from the FAO using outdated sources from the years 1964–2001. Nevertheless, even if greenhouse gas emissions are estimated at only 18%, the livestock industry is still the second-largest polluter after the electricity industry, and more polluting than the transportation industry, which contributes approximately 13% [6].

Most emissions related to the livestock industry are in the form of carbon dioxide (CO_2_), nitrous oxide (N_2_O), methane (CH_4_), and ammonia (NH_3_) [6,7]. Domestic animals ‘naturally’ release carbon dioxide, which has been proven to be a significant contributor to global warming [5]. Researchers warn that we will probably exceed the 565 gigaton carbon dioxide limit by the year 2030 due to livestock rearing. In addition, the livestock industry is responsible for 68% of enterogenic nitrous oxide emissions; this gas remains in the atmosphere for up to 150 years and has a 296-fold greater potential for global warming and deterioration of the ozone layer than carbon dioxide. Livestock emit almost 64% of total ammonia emissions, contributing significantly to acid rain and to acidification of ecosystems. Livestock are also a highly significant source of methane emissions, contributing 35–40% of methane emissions worldwide. Methane has a 23-fold greater potential for global warming than carbon dioxide. The U.S. Environmental Protection Agency has shown that in the last 15 years methane emissions from pigs increased by 37% and emissions from cattle increased by 50% [6,7].

Secondly, while not all livestock impacts environment in the same way, production of animal products might require extensive land. Farms for rearing livestock already cover one-third of the world’s total land and more than two-thirds of its agricultural land [3]. The increasing demand for animal products and the lack of land has caused the livestock industry to become the main cause for clearing forests and turning them into pasture. According to the International Center for Forest Research (CIFOR), during the years 1990–2000, an area twice the size of Portugal was lost in favor of pasture [3]. Another reason for forest clearing is production of food for animals. Approximately 40% of the harvested crops in the world are used as food for animals. Thus, if we took half of the crops used as feed for those same animals, we would be able to feed all the starving populations around the world and solve the problem of world hunger [3,8]. Massive forest clearing leads, among other things, to animal extinctions [9]. Up to 137 species of plants, animals and unique insects are lost every day due to forest clearing. Ceballos et al. [10] claim that this is the greatest mass extinction in 65 million years. While it is well documented that the livestock industry and livestock production cause a host of environmental problems, livestock production in certain ecosystems, like arid and semi-arid lands, are the most well-adapted food production system. It is a more efficient and rational land use system (if animals are able to move) than cultivation, which has a poorer track record at feeding people and being sustainable, especially under conditions of increasing climatic variability. Farming some staple crops, like rice, also has extensive negative environmental impacts [11,12,13].

Production of animal food products is the greatest agricultural cause of water pollution [3]. The trend of increasing consumption of animal products has a negative impact on ecosystems and on water sources, in particular in developing countries. The water pollution is caused by animal excreta, antibiotics and hormones, fertilizers and pesticides used in forage production, and rainfall runoff from pasture [1]. The U.S. Department of Agriculture (USDA) declared that animal parts and poultry manure are major sources of water pollution [3].

The livestock industry also leads to great resource wastage, in particular wastage of water [1]. In the U.S., for example, the amount of water consumed by private residences is approximately 5% of total consumption, while the amount of water consumed by animal agriculture is approximately 55% [14]. A study that measured the amount of consumed resources (e.g., water, fertilizer, soil) and greenhouse gas emissions from food showed that a vegan diet is better for reducing environmental impacts compared to a vegetarian or omnivorous diet [15]. In one day, a vegan person saves 4164 liters of water, 20 kg crops, 2.8 m^2^ forested land, 10 kg CO_2_ and the life of one animal [16].

Moreover, the livestock industry produces copious quantities of waste. The livestock industry in the U.S. produces 116,000 pounds of waste per second. According to Haines and Staley [17] a farm with 2500 milking cows produces the same amount of waste as a city with 411,000 residents. Thus, we must ask whether the general population is aware of these damages caused by the livestock industry to the environment.

### 1.3. People’s Level of Awareness of Environmental Pollution Caused by the Livestock Industry

Environmental problems, particularly climate change resulting from human activities, continue to hold a prominent place on the international agenda [18]. While the general population is aware of environmental problems such as air or water pollution it is barely aware of the environmental damages caused by the food industry. Consumers are less aware of the impact of their food choices, through production and food distribution, than of other popular issues, such as industrial pollution and wildlife conservation [19]. Awareness is particularly low with respect to environmental pollution by the livestock industry. Despite the high awareness of consumers about the health benefits of reducing meat consumption, the environmental impacts of reducing consumption are barely known. A number of studies conducted in Europe showed that consumers may be concerned about animal food production, but their knowledge on this issue is very minimal and often comes from unreliable sources, and thus many continue to consume animal products [20].

In a study that examined the behavior and beliefs of consumers in Australia with respect to food, 223 participants were asked to rank the most important food-related activities for conserving environmental quality. ‘Reducing plastic bags’ and ‘compost’ were found to be the most important activities while ‘reducing meat consumption’ was considered by consumers to be the activity with the lowest impact on environmental quality [21].

Consumer attitudes towards pork consumption were examined in a study that combined the findings from two European Union projects [20,22]. One project included eight focus groups with seven to nine participants in each group. In total, 65 people aged nineteen to sixty from the capital cities of Germany, France, Spain and Britain took part in the discussions. All participants were meat eaters who consumed pork at a frequency of ‘at least once a week’ to ‘nearly every day.’ The discussions were intended to extract information on the participants’ opinions and attitudes towards eating meat, safety, and health. In the second project, data were collected via an online survey conducted among 2437 people aged twenty to seventy in five countries: Belgium, Germany, Poland, Greece and Denmark. The data included socio-demographic information about the participants, weight and height, attitudes, and information about behavior related to meat consumption. With respect to attitudes, heavy pork consumers supported large-scale pork production systems. ‘Intermediate frequency, high diversity’ consumers were considered to be more ‘environmentally conscious’ that all of the other groups. Their low meat consumption in comparison to the heavy consumers may be related to their attitudes towards the environmental consequences of pork production. Rare consumers of pig (‘low frequency, low diversity’) were considered to be more concerned about animal well-being and supported small pork production systems. As a rule, it was found that on average across the entire sample, attitudes towards environmental quality and animal food production were very weak. Even the consumers who expressed concern for the environment with respect to pork production continued to consume it on a daily basis. Similarly, consumers who indicated that they do not eat pork at all did not avoid it because of environmental concerns but rather due to other reasons.

A long-term study conducted in Switzerland among 6189 participants (47% males) examined eating habits and aspects related to nutrition and food consumption [23]. The project lasted one year and studied how people’s food consumption patterns change with time and which factors are related to these changes. The results of the study showed that the consumers believed that ‘avoiding food with excessive packaging’ would have a beneficial impact on the environment. In contrast, they ranked the option of ‘avoiding meat’ as being the least beneficial to the environment. The more meat the participants ate, the more negative their attitude towards the benefit of reducing meat consumption. Since it is difficult for consumers to give up meat, denying the benefit of reducing meat consumption may be their strategy for reducing the dissonance but may also reflects a lack of knowledge. With respect to reducing meat consumption and buying organic food, most participants were not willing to make any change and were in the pre-contemplation stage. Women were more willing to reduce consumption or had already reduced meat consumption (meaning, they were in the active stage) compared to men. People who believed that reducing meat consumption has a positive impact on their health ate less meat. Conversely, participants who believed that reducing meat consumption has a positive impact on the environment reflected this less through their behavior. Similarly, the ethical aspect of cruelty to animals only affected the willingness of consumers to consider reducing meat consumption but not to progress to the active stage. It was also found that for all consumption patterns, women are more ‘environmentally friendly’ than men. The difference was most marked with respect to purchasing organic food. In addition, men were significantly less willing to reduce their meat consumption.

Due to the low awareness found in countries around the world, it is of paramount importance to examine the knowledge, attitudes and behavior of consumers with respect to the environmental consequences of the meat industry. A better understanding of knowledge, attitudes and behavior of consumers might serve to improve the current debate on the impact of livestock industry on environment and health. 

### 1.4. The Relationship between Knowledge, Attitudes, and Pro-Environmental Behavior

Knowledge, as a cognitive component, is indeed critical, but alone it cannot adequately predict pro-environmental behavior. The emotional component, which is related to attitudes and values, is essential for driving the transformation of knowledge to responsible environmental behavior [24]. Despite the complex relationship between the components, researchers have shown that expanding knowledge via environmental studies and educational activities leads to more positive attitudes towards the environment and more responsible environmental behavior [25,26].

Pe’er et al. [24] examined the level of environmental literacy of 765 students studying teaching at three teachers’ colleges in Israel. It was found that the students had low ecological-environmental knowledge (38.39 out of 100, on average), but most of them expressed positive attitudes (3.59–4.13 on a scale of 1–5). The Pearson correlation coefficients showed a high correlation between attitudes and behavior (*r* = 0.49, *p* < 0.001) and a low correlation between knowledge and behavior (*r* = 0.23, *p* < 0.01).

Tuncer et al. [27] examined the relationship between knowledge, attitudes, and concern for the environment among 684 teachers in Turkey. Half of the respondents (51%) defined themselves as ‘quite concerned’ and only 11% reported a high level of concern for environmental problems. The participants did not express high confidence in their level of environmental knowledge, with less than 4% reporting that they were ‘quite proficient’ on environmental issues, and 55% of them having ‘some kind of environmental knowledge’. Despite the poor knowledge, the teachers’ attitudes, on average, were positive towards the environment and their view was considered to be an ecological world view. The researchers found positive relationships between the level of knowledge and the level of concern for the environment (*r* = 0.13, *p* < 0.01) and between environmental attitudes and level of concern (*r* = 0.20, *p* < 0.01).

In summary, increasing knowledge, skills, approaches and values within the individual with respect to the environment may promote the individual’s feeling of responsibility and capability to change his/her behavior to be more pro-environmental. Nevertheless, studies show that even when a person prides themselves on particular values, in many cases he/she does not act to implement them. This is the gap between declared values and actual decisions [28]. In particular, in the environmental field there is a gap between the social and environmental values that a person believes in and his/her consumer behavior; this is known as the value-action gap [29]. An example of this was found in a survey conducted in the U.S., which found that 40% of consumers hold positive opinions about ‘green’ products but in practice they do not purchase them due to a number of reasons (cost, accessibility, convenience) [30].

### 1.5. The Relationship between Animal Rearing and Knowledge Levels, Attitudes, and Behavior

The relationship between rearing pets and empathy towards animals has been examined by a number of studies. Paul [31] found that empathy towards animals was significantly related to present or past ownership of pets. In a sample of 514 adolescents in Scotland, it was found that children and young adults who reared pets loved farm animals and wild animals more than children who grew up without pets [32]. In addition, a number of studies have shown that pet owners demonstrate more empathy towards animals and show greater opposition to cruelty towards them [33,34].

Meat consumption is also related to attitudes towards animals. For example, it has been found that the main reason for vegetarian nutrition is animal welfare [35,36]. In a survey of students, Paul and Serpell [37] found that as the reported number of animals that were important to the respondent in some way during his/her childhood increased, the student was more likely to report avoidance of at least one animal product for ethical reasons. In a qualitative study in which 11 vegetarians were interviewed, most of the interviewees related vegetarianism during adulthood to ownership of pets during their childhood [38]. In another study, vegetarian males related more positively to pets than non-vegetarian males [39]. Moreover, a number of studies have reported a higher proportion of pet owners among meat-avoiders [40]. As a rule, it seems that perception of the environment is also affected by attitudes towards animals. Pifer, Shimizu and Pifer [41] found a significant relationship between concern for the environment and opposition to experiments on animals and concern for their rights in eleven out of fifteen countries.

From this literature review we can appreciate the destructive impact of the livestock industry on various, diverse aspects of the environment. Due to increased global trade in animal products, crop production for animals, and long-term meat preservation, it seems that consumers have become spatially disconnected from the necessary processes involved in production of animal products [42]. They do not connect food products and environmental quality; and they are barely aware of the environmental impact of the consumption of animal products [23]. The aim of this current study is to examine the level of knowledge and awareness of students in Israel on topics related to environmental pollution caused by industrial animal food production. Similarly, the study aspires to examine the behavior of participants with respect to this issue, and to determine whether there is a relationship between knowledge, attitudes, and behavior. The research hypothesis is that positive relationships will be found between the level of knowledge, attitudes, and behavior on topics related to environmental pollution caused by the livestock industry, whereby attitudes will mediate the relationship between the level of knowledge and behavior. In addition, participants who own pets or owned them in the past will demonstrate greater knowledge, awareness, and pro-environmental behavior than other participants.

## 2. Materials and Methods

### 2.1. Study Population and Sample

The study was conducted among students enrolled at Ashkelon Academic College in 2017. According to data from the Higher Education Council (HEC), 3453 students studied at the college during that year, including 70% women. The sample comprised of 361 students who answered at least 80% of the questionnaire; they comprised 11% of the total number of students at the college. Responding to the questionnaire indicated informed consent to participate in the survey. There were no exclusion criteria for the study.

### 2.2. Research Tools

For the current study, we used an anonymous, closed, self-completion questionnaire. We did not find questionnaires that examined the variables in the current study and a new questionnaire was therefore constructed. For this purpose, we conducted an extensive literature review. Since there were no similar previous questionnaire testing knowledge on livestock industry influence on the environment (apart from surveys dealing with pork industry that is not relevant to the Israeli context). The questionnaire was validated by sustainability experts using a content validation method. Subsequently, a pilot study was conducted among 10 students who do not study at Ashkelon Academic College, and two unclear questions were corrected.

#### Description of Questionnaire Sections:

The questionnaire comprised 46 closed questions as follows:

1. Demographic information—six questions about gender, age, marital status, country of birth, nutritional lifestyle (omnivore/vegetarian/vegan), and whether the respondent previously or currently rears animals.
2. Knowledge—thirteen questions in which respondents were asked to indicate whether, in their opinion, the statement is correct or incorrect or whether they do not know. For example: The livestock industry causes more environmental pollution than the transportation industry. Questionnaire reliability: Cronbach’s α = 0.90.
3. Attitudes—thirteen questions relating to attitudes towards the livestock industry in which respondents were asked to indicate to what extent they agree with the statement on a Likert scale of 1–5, including the option “I don’t know”. For example: It is important to me that the food I eat is produced in a way that preserves animal rights. Questionnaire reliability: Cronbach’s α = 0.88.
4. Behavior—seven questions. Respondents were asked to indicate at what frequency they act according to the statement on a Likert scale of 1–5, including the option “I don’t know.” For example: I participate in the battle to prevent hazards from the livestock industry. Questionnaire reliability: Cronbach’s α = 0.71.
5. Consumption of animal products—respondents were asked to indicate at what frequency they consume beef, chicken, fish, eggs, dairy products, organic vegetables and meat substitutes on a scale ranging from 1 (not at all) to 5 (every day).

### 2.3. Research Process

This study was a cross-sectional study. In the first stage we conducted an extensive literature review for the purpose of constructing and validating the questionnaire. After approval from the ethics committee of the college, the questionnaires were programmed using Qualtrics and distributed to the students in March 2017. A reminder to complete the questionnaire was sent in the same way after two weeks. On 5 April 2017, the questionnaire was closed in the program. The time taken to answer the questionnaire was estimated at seven minutes on average. There were 541 entries to the questionnaire, and 361 students completed at least 80% of the questionnaire (67% of those entering the questionnaire), thus 180 participants were omitted from the analysis.

The introductory page to the questionnaire contained an explanation of the essence and aim of the questionnaire. Completion of the questionnaire indicated informed consent to participate in the survey and the students could stop responding to it at any stage or to choose not to answer some of the questions. No questions were defined as compulsory.

### 2.4. Data Analysis

The data were analyzed using SPSS V. 23 (IBM, Armonk, NY, USA). The relationships between the variables were examined by calculating Pearson correlations. Mediation was examined using linear regressions according to Baron and Kenny [43]. Differences between groups were examined using independent *t*-tests. Finally, hierarchical (multiple) linear regression models were built to predict pro-environmental behavior, with gender and rearing animals as covariables. The model included variables that were found to be significantly related to behavior in the univariate analyses.

## 3. Results

### 3.1. Description of Sample Characteristics

The study participants included 361 students aged 18 to 67; the average age was 29 (SD = 8.6). The sample characteristics are presented in Table 1.

Table 1 shows that most participants were women (75%), similar to the percentage of female students in the general student population at the college (77%). Most participants were born in Israel (77%) and omnivorous (91%). Half of them are single (49%) and 46% are in a relationship. More than half of them rear or previously reared an animal (55%). Two thirds study in the Faculty of Social Sciences, 15% in the Faculty of Health Sciences (psychology, sociology, criminology, social work, etc.), 11% in the Faculty of Engineering, and 8% in the Faculty of Management.

### 3.2. Level of Knowledge

The distribution of responses to the statements that examined the level of knowledge with respect to environmental damage cause by the livestock industry is presented below (Table 2).

To construct the variable “level of knowledge about the damages caused to the environment by the livestock industry”, we counted the number of correct answers provided by each participant. The variable ranged from 0–13. The mean value of the knowledge variable was 3.33 (SD = 2.38).

### 3.3. Attitudes

The distribution of responses to statements that examined attitudes are presented below (Table 3) after combining categories as follows: answers 1 and 2 were combined into the category ‘weakly agree,’ answer 3 remained ‘moderately agree’ and answers 4 and 5 were combined into the category ‘strongly agree’.

For the purpose of constructing the attitudes variable we calculated the mean response of each participant, without the ‘I don’t know’ option, and after reversing the scale for questions 9 and 12. The mean value of the variable was 3.28 (SD = 0.80).

### 3.4. Behavior

The distribution of responses to the statements, after combining categories, is presented below (Table 4).

For the purpose of constructing the variable we calculated the mean response for each participant, without the ‘I don’t know’ option. The mean value of the behavior variable was 2.41 (SD = 0.71).

### 3.5. The Relationships between Knowledge, Attitudes, and Behavior

We found positive and strongly significant relationships between level of knowledge and attitudes (*r* = 0.33, *p* < 0.001), between level of knowledge and behavior (*r* = 0.36, *p* < 0.001), and between attitudes and behavior (*r* = 0.49, *p* < 0.001). In other words, the higher the level of knowledge, the more pro-environmental were the attitudes and behavior. More pro-environmental attitudes were related to more pro-environmental behavior. Therefore, the hypotheses are confirmed.

### 3.6. Attitudes Mediating the Relationship between Knowledge and Behavior

**Figure 1:**
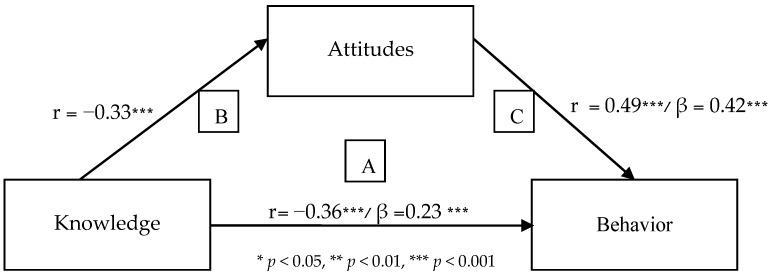
Attitudes mediate the relationship between knowledge and behavior.

According to the method of Baron and Kenny [44], three linear regressions were performed (Figure 1): firstly, we examined the predictive ability of knowledge on behavior (A). Secondly, we examined the predictive ability of knowledge on attitudes (B). Thirdly, knowledge and attitudes were included as independent variables, with behavior as the dependent variable (C). As shown in Figure 1, in the first regression (path A) we found that the knowledge variable predicted behavior (*β* = 0.36, *p* < 0.001), explaining 13% of variance in behavior. In the second regression (path B) we found that the knowledge variable predicted attitude (*β* = 0.33, *p* < 0.001), explaining 11% of variance in attitudes. In the third regression (path C) we found that the knowledge and attitude variables explained 28% of variance in the behavior variable. When we added the attitude variable, the amount of variance explained increased to 23% and the power of the corrected regression coefficient (*β*) of the knowledge variable decreased (*β* = 0.23, *p* < 0.001). The attitude variable was found to significantly predict behavior (*β* = 0.42, *p* > 0.001), thus we can conclude, according to Baron and Kenny [44] that the attitude variable partly mediates the relationship between knowledge and behavior. In other words, if we controlled for the effect of attitude, there was still a relationship between knowledge and behavior, but it was weaker. Similarly, the change in the percent variance explained was significant (R^2^ change = 0.29, *p* < 0.001), therefore, confirming our hypothesis.

### 3.7. Rearing Animals

Significant differences were found between participants who rear/reared animals and participants who do not/did not, in the level of knowledge (*t*_(355)_ = 3.78, *p* < 0.001), attitudes (*t*_(354)_ = 3.04, *p* < 0.01), and behavior (*t*_(329)_ = 2.33, *p* < 0.05) on issues related to environmental pollution caused by the livestock industry. Participants who rear/reared animals had more knowledge (mean = 3.29 vs. 2.62 among participants who do not rear animals), more positive attitudes (mean = 3.40 vs. 3.14 among participants who do not rear animals), and more pro-environmental behavior (mean = 2.49 vs. 2.30 among participants who do not rear animals), therefore, confirming our hypothesis.

### 3.8. Differences between Genders

No differences were found between genders in the level of knowledge, but significant differences were found between genders with respect to attitudes (*t*_(354)_ = 2.45, *p* < 0.05) and behavior on topics related to environmental pollution caused by the livestock industry (*t*_(333)_ = 3.26, *p* = 0.001). Women had more positive attitudes (mean = 3.34 vs. 3.10) and pro-environmental behavior (mean = 2.47 vs. 2.20) than men.

### 3.9. A Linear Regression Model to Predict Pro-Environmental Behavior

The results of the hierarchical (multiple) linear regression models to predict pro-environmental behavior, where gender and rearing animals were covariables, are presented below (Table 5). The models included variables that were significantly related to behavior in the univariate analyses.

In the final model, which included all of the variables that were significant in the previous models, the ability of all variables to predict pro-environmental behavior was maintained. It is clear that attitudes were the best predictor of behavior (*β* = 0.28, *p* < 0.001). They were followed by beef consumption (*β* = −0.25, *p* < 0.001) and meat substitutes (*β* = 0.19, *p* < 0.001). The combined model shows that knowledge, consumption of milk products, organic vegetables and eggs also predicted behavior (*β* = 0.14, *p* < 0.01; *β* = −0.12, *p* < 0.01; *β* = 0.12, *p* < 0.01; *β* = −0.10, *p* < 0.5, respectively). The variance explained by the final model was approximately 44% (*p* < 0.001).

## 4. Discussion

The present study examined the level of knowledge, attitudes and behavior of students on topics related to environmental pollution caused by the livestock industry. It was found that participants’ attitudes towards damage caused to the environment by the livestock industry are moderately pro-environmental, and the level of knowledge on the subject is low. Moreover, students do not demonstrate pro-environmental behavior in this context. These findings are in line with a number of studies conducted in Europe and the U.S., which showed that some consumers are concerned about production of animal foods but their knowledge on this topic is very limited, and most continue to consume animal products without any intention of reducing consumption [20,21,22,45].

The greatest strength in this relationship was found between attitudes and behavior, followed by the relationship between level of knowledge and behavior and finally between level of knowledge and attitudes. In recent years, environmental issues have attained an increasingly significant place on the media’s agenda. Studies in environmental education have found a clear relationship between acquiring knowledge during an educational activity and an increase in positive attitudes towards the environment [20,26,27,46]. Many studies have strengthened this finding and shown that environmental knowledge is needed to drive responsible environmental behavior, and that it is a prerequisite for action [28,47,48]. The survey conducted by Rickinson [49] also showed that environmental knowledge is indeed an important component in the prevalence of supportive environmental behavior and is a prerequisite for formulating attitudes towards environmental problems. However, knowledge is not the central component affecting behavior [25]; indeed, the findings of the present study show that the strength of the relationship between attitudes and behavior is greater than the strength of the relationship between knowledge and behavior.

It was also found that attitudes partially mediate the relationship between the level of knowledge and behavior. In other words, if we account for the effect of attitudes, there will still be a relationship between knowledge and behavior, but it will be weaker. According to Pe’er et al. [25], knowledge is indeed critical but knowledge alone cannot adequately predict responsible environmental behavior. The emotional component, which is related to attitudes and values, is necessary for driving the transformation of knowledge into responsible environmental behavior. In other words, the environmental behavior of the individual may change due to changes in values, beliefs and pro-environmental norms. The theory of reasoned action (TRA) of Fishbein and Aizen [29], which connects beliefs, attitudes, intentions, and behavior, can provide an explanation for this finding. Fishbein and Aizen claimed that the intention to conduct behavior is the best predictor of its occurrence, and it depends on the attitudes and norms held by the individual. The individual’s knowledge and positive attitudes, alongside social norms that call for environmental conservation, will create a socialization process that strengthens environmental values. These will create motivation and intentions to act to reduce damages caused to the environment by the livestock industry.

As hypothesized, it was found that participants that rear/reared animals demonstrated more knowledge, attitudes, and pro-environmental behavior than others. These findings are supported by a number of studies showing that pet owners demonstrate more empathy towards animals and greater opposition to cruelty towards them [32,34,35]. In addition, in some studies the proportion of pet owners was higher among a group of meat-avoiders [39,41] and that the main cause of vegetarian nutrition was animal welfare [36,37,40].

The study did not find differences between genders in the level of knowledge, but nevertheless significant differences between genders were found for attitudes and behavior. Women had more positive attitudes and pro-environmental behavior than men. Dietz et al. [50] reported similar findings, and explained that in their opinion, parenthood leads to greater environmental concern among women than among men. Stern et al. [51] found that women expressed more positive attitudes towards environmental quality, stronger intentions regarding the need for pro-environmental behavior, and stronger opinions about the destructive consequences of deteriorating environmental quality, than men. Tobler et al. [24] found that women were much more willing than men to give up meat. The authors offered the explanation that meat, and in particular red meat, is linked to strength and power, which makes it difficult for males to change their attitudes and reduce their consumption.

Finally, a hierarchical (multiple) linear regression model was built to predict pro-environmental behavior, wherein gender and animal rearing were covariates. The model included variables that were found to be significantly related to behavior in the univariate analyses. In the final model it was found that knowledge, attitudes, consumption of beef and dairy products (inversely correlated), meat substitutes, organic vegetables, and eggs predict pro-environmental behavior. The explained variance of the final model was 44%.

If this is the case, environmental behavior is a function of increasing knowledge, sensitivity, skills, approaches and values held by the individual towards the environment. Nevertheless, there is sometimes a gap between social and environmental values that a person aspires to believe in and his/her consumer conduct [30], as was also shown in the study by [24]. A possible reason for this could be that many people do not know what to do in order to behave in a pro-environmental way or that pro-environmental behavior involves a conflict between the individual’s immediate need to the long-term environmental interest [52]. Preferring the present over the future is a ‘classic’ sustainability problem, since intentional sustainable behavior necessitates long-term thinking and giving preference to future benefits over present, short-term benefits [53].

### 4.1. Limitations of the Study

The present study was conducted only at Ashkelon Academic College, and may not be a representative sample. The study is a cross-sectional study, and due to a lack of means, other factors linked to pro-environmental behavior were not examined. Similarly, the research questionnaire written by the researchers (following validity by experts) was used for the first time in this study. It is possible that the knowledge questions were difficult, and putting them at the beginning of the online questionnaire may have deterred participants (approximately 150 students stopped filling out the questionnaire after the knowledge questions). Another limitation of the study may be the social desirability bias of the participants. Meaning, participants may have marked answers they thought the researchers wanted to receive. Finally, the study used an online questionnaire, and it may be that the subject was of concern for those who participated, creating a selection bias. We assume that since the average knowledge, attitudes and behavior were relatively low, these last two limitations did not lead to significant bias in the results, if at all.

### 4.2. Recommendations

Students have almost no knowledge about the environmental impacts of the food they consume, and in particular, animal products, indicating that campaigns to raise awareness of this issue are likely to be effective, especially since we found that knowledge is positively related to attitudes and behavior. We recommend including an introductory course in environmental studies (from the perspective of climate change and the relationship between health and the environment) in the study programs of all departments, with an emphasis on health subjects. Moreover, this issue is not adequately emphasized in public health schools in Israel; indeed, discussion of the impacts of the livestock industry is fundamental due to aspects related to human nutrition as well as aspects related to the many damages caused by this industry to the environment, as described in this study.

Future research to examine the level of knowledge, attitudes, and behavior needs to be conducted on a representative sample of other populations, such as school children, adult populations, health and medical professionals, and more. A more in-depth study could include focus groups and interviews in to better examine the knowledge and awareness of consumers with respect to food choices.

## 5. Conclusions

In this study we found that students have almost no knowledge about the environmental impact of the food they consume, their attitudes are moderately pro-environmental yet they are not strict about pro-environmental behavior. Students with higher levels of environmental knowledge demonstrated more pro-environmental attitudes and behavior; attitudes mediate the relationship between level of knowledge and behavior with respect to environmental pollution caused by the livestock industry. In addition, participants that rear/reared animals demonstrated more knowledge and pro-environmental attitudes and behavior, and women demonstrated more pro-environmental attitudes and behavior than men.

Future campaigns on environmental education should place emphasis on the contribution of the individual to impacts on the environment, consumer habits relevant to the environment and the environmental and health benefits of consuming plant-based foods and organic food. Agriculture, and in particular animal husbandry, produces significant pollution and it will be possible to influence consumer’s food choices if they understand the environmental impacts of the livestock industry. Reducing consumption of animal products will probably be promoted most effectively by describing the health benefits of this action, as well as the ethical aspects of preventing cruelty to animals.

Different initiatives around the world are now being promoted, such as Meatless Monday, increasing awareness to nutritional values found in other products than livestock industry products and awareness campaigns. All these practices should be evaluated in order to promote best practices to tackle this pressing issue.

## Figures and Tables

**Table 1:** Description of sample characteristics (*n* = 361).

Character	*n*	%
Males	91	25
Single	176	49
Married, live with partner	165	46
Divorced/separated	16	5
Born in Israel	276	77
Born overseas	85	23
Omnivore	328	91
Vegetarian/vegan	33	9
Rear/reared an animal	198	55
Humanities and Social Sciences	237	66
Health Sciences	53	15
Engineering	37	11
Management	30	8

**Table 2:** Distribution of responses to the knowledge questionnaire.

Statement	Correct (%)	Incorrect (%)	Don’t Know (%)
1. The increase in consumption of meat products contributes directly to climate change.	35	17	48
2. Fertilization and soil waste produce about two-thirds of all agricultural emissions around the world.	28	5	67
3. About 20% of global greenhouse gas emissions are from the livestock industry.	32	12	56
4. The livestock industry is the second greatest polluter after the electricity industry.	22	22	56
5. The livestock industry causes greater environmental pollution than the transportation industry.	12	39	49
6. The average amount of water consumed by private homes is estimated at about 5%, while the amount of water consumed by animal agriculture is about 55%.	26	13	61
7. The amount of water required to produce 1 kg meat is at least 50 times greater than the amount of water required for vegetable production.	22	15	63
8. About 40% of crops harvested around the world are used as food for animals.	36	8	56
9. Exposure to organic fertilizer in drinking water and vegetables is a risk factor for cancer.	31	11	57
10. About 2.7 trillion marine animals are drawn from the oceans each year.	30	6	64
11. Livestock production takes up 70% of all agricultural land.	21	18	61
12. Livestock production takes up 30% of the earth’s land.	25	11	64
13. The livestock industry is responsible for about 90% of rainforest destruction.	13	27	60

**Table 3:** Distribution of responses to the attitudes questionnaire.

Statement	Weakly (%)	Moderately (%)	Strongly (%)	Don’t Know (%)	Mean ± SD ^1^
1. The livestock industry causes environmental destruction.	25	23	30	22	3.12 ± 1.31
2. The vegan diet is the best one for reducing the environmental impact of the livestock industry.	42	13	27	18	2.64 ± 1.51
3. The livestock industry leads to great wastage of natural resources (water, food, land).	34	20	28	18	2.90 ± 1.39
4. The production of animal products should be limited.	39	20	30	11	2.83 ± 1.42
5. It is important to me that the food I eat is produced in an environmentally friendly way.	15	20	61	4	3.81 ± 1.25
6. It is important to me that the food I eat is produced in a way that preserves animal rights.	14	20	62	4	3.92 ± 1.20
7. The issue of environmental destruction by the livestock industry should be much higher on Israel’s list of priorities.	18	26	51	6	3.54 ± 1.22
8. It is very important to me to preserve environmental quality.	8	16	73	3	4.10 ± 1.03
9. Plants and animals exist so that humans will use them for their needs. *	35	21	39	5	2.11 ± 1.41
10. If had more knowledge on the issue, I am sure that I would integrate environmental considerations when choosing my food.	21	22	50	7	3.50 ± 1.27
11. The livestock industry should be obligated to reduce polluting emissions to the environment even if this means that the cost for the consumer will rise.	26	24	41	9	3.30 ± 1.35
12. The issue of concern for environmental problems is exaggerated. *	56	21	16	7	1.28 ± 1.27
13. Every student should be obligated to participate in a course on environmental issues during his/her degree.	56	15	23	6	2.39 ± 1.45

^1^ The mean was calculated without including the ‘I don’t know’ option. * Opposite questions. The data are presented in reverse rank order.

**Table 4:** Distribution of responses to the behavior questionnaire.

Statement	Rarely (%)	Sometimes (%)	Often (%)	Don’t Know (%)	Mean ± SD ^1^
1. I buy food made in Israel.	6	23	63	8	4.01 ± 0.99
2. I eat food according to the season.	26	24	47	3	3.30 ± 1.37
3. I eat organic food.	65	23	9	3	2.08 ± 1.07
4. I am considering becoming vegetarian or vegan.	74	9	15	2	1.87 ± 1.32
5. I try to consume food from the livestock industry as little as possible.	59	20	20	1	2.32 ± 1.40
6. I participate in the battle to prevent hazards from the livestock industry.	90	4	4	2	1.33 ± 0.82
7. I read articles on hazards from the livestock industry.	65	19	15	1	2.05 ± 1.23

^1^ The mean was calculated without including the ‘I don’t know’ option.

**Table 5:** Results of hierarchical linear regression models to predict pro-environmental behavior.

Variable	Background Variables	Knowledge and Attitudes	Consuming Animal Products	Combined Model
	*β*	*β*	*β*	*β*
Gender (0—male, 1—female)	0.15 **	0.09	0.04	
Rearing animals (0—no, 1—yes)	0.11 *	0.01	0.12 **	
Knowledge		0.23 **		0.14 **
Attitudes		0.41 **		0.28 **
Beef			−0.30 ***	−0.25 ***
Poultry			−0.09	
Fish			0.02	
Eggs			−0.12 *	−0.10 *
Dairy products			−0.13 **	−0.12 **
Organic vegetables			0.15 **	0.12 **
Meat substitutes			0.22 ***	0.19 ***
Adjusted R Square	0.03 **	0.29 ***	0.36 ***	0.44 ***
*n*	335	332	323	321

* *p* < 0.05, ** *p* < 0.01, *** *p* < 0.001.
